# Mechanisms of mutant β-catenin in endometrial cancer progression

**DOI:** 10.3389/fonc.2022.1009345

**Published:** 2022-09-29

**Authors:** Molly L. Parrish, Russell R. Broaddus, Andrew B. Gladden

**Affiliations:** ^1^ Department of Pathology and Laboratory Medicine, The University of North Carolina at Chapel Hill, Chapel Hill, NC, United States; ^2^ Pathobiology and Translational Science Graduate Program, The University of North Carolina at Chapel Hill, Chapel Hill, NC, United States

**Keywords:** endometrial cancer, β-catenin, tumor progression, cell adhesion, Wnt-signaling

## Abstract

Endometrial carcinoma (EC) is the most diagnosed gynecological malignancy in Western countries. Both incidence and mortality rates of EC have steadily risen in recent years. Despite generally favorable prognoses for patients with the endometrioid type of EC, a subset of patients has been identified with decreased progression-free survival. Patients in this group are distinguished from other endometrioid EC patients by the presence of exon 3 hotspot mutations in *CTNNB1*, the gene encoding for the β-catenin protein. β-catenin is an evolutionarily conserved protein with critical functions in both adherens junctions and Wnt-signaling. The exact mechanism by which exon 3 *CTNNB1* mutations drive EC progression is not well understood. Further, the potential contribution of mutant β-catenin to adherens junctions’ integrity is not known. Additionally, the magnitude of worsened progression-free survival in patients with *CTNNB1* mutations is context dependent, and therefore the importance of this subset of patients can be obscured by improper categorization. This review will examine the history and functions of β-catenin, how these functions may change and drive EC progression in *CTNNB1* mutant patients, and the importance of this patient group in the broader context of the disease.

## Introduction

Endometrial carcinoma (EC) is the most common gynecological cancer in the industrialized world. EC poses a unique challenge, as incidence and mortality rates continue to rise despite many other major cancer types declining in recent years (1). An estimated 65,950 new cases and 12,550 deaths are predicted to arise in 2022. Strikingly, endometrial cancer mortality likelihood has risen from a 0.3% chance from 1997-2008 to a 1.9% chance from 2008-2018 (1). Endometrial cancer is primarily defined by two histological subtypes, endometrioid and non-endometrioid, where endometrioid EC comprises approximately 80% of cases (2). Endometrioid endometrial cancers (EEC) are characterized by being low grade and having glandular histology resembling the normal endometrium. EEC is associated with intrinsic risk factors like obesity and excess estrogen and presents at a low stage at the time of diagnosis. Further, endometrial hyperplasia can be a precursor for EECs. Conversely, non-endometrioid endometrial cancers are far less common and not typically associated with environmental or life-style related risk factors. These cancers tend to be more aggressive, presenting with higher stage and higher grade non-endometrioid histological types including serous and clear cell histology.

Endometrioid and non-endometrioid endometrial cancers have different mutational signatures. Endometrioid cancers have a broader mutational spectrum than non-endometrioid cancers. Alterations in the PI3K/Akt/mTOR pathway, Wnt/β-catenin pathway, and mismatch repair genes are common in EEC while *TP53* mutations dominate non-EECs (3). Within the PI3K/Akt/mTOR pathway, the negative regulator *PTEN* is frequently mutated, as well as *PIK3CA*, the catalytic PI3K subunit. In addition to mutations in the PI3K/Akt/mTOR pathway, a subset of endometrioid cancers also harbor mutations in *CTNNB1*, which encodes for the Wnt-signaling protein β-catenin. More broadly, endometrial cancer can be divided into four molecular subtypes, as determined by The Cancer Genome Atlas: POLE (ultramutated), MSI-high (hypermutated), Copy-number low, and Copy-number high (4). The POLE, MSI-high, and Copy-number low molecular subtypes most often correspond with an endometrioid histology. Conversely, the Copy-number high molecular subtype are nearly all serous histology. Endometrioid cases with *CTNNB1* mutations are most often classified as Copy-number low, though some are classified as MSI-high.

Mutations in *CTNNB1* occur early in endometrial cancer pathogenesis, as evidenced by β-catenin dysregulation in atypical hyperplasia (5, 6). Further deletion of exon 3 of *CTNNB1* in a murine model drives endometrial hyperplasia (7). Additional investigations found that *PTEN* loss coupled with activation of β-catenin through Wnt-signaling led to an earlier onset and more aggressive endometrial cancer in a mouse model (8). This suggests *CTNNB1* mutations may drive endometrial cancer progression.

**Figure 1:**
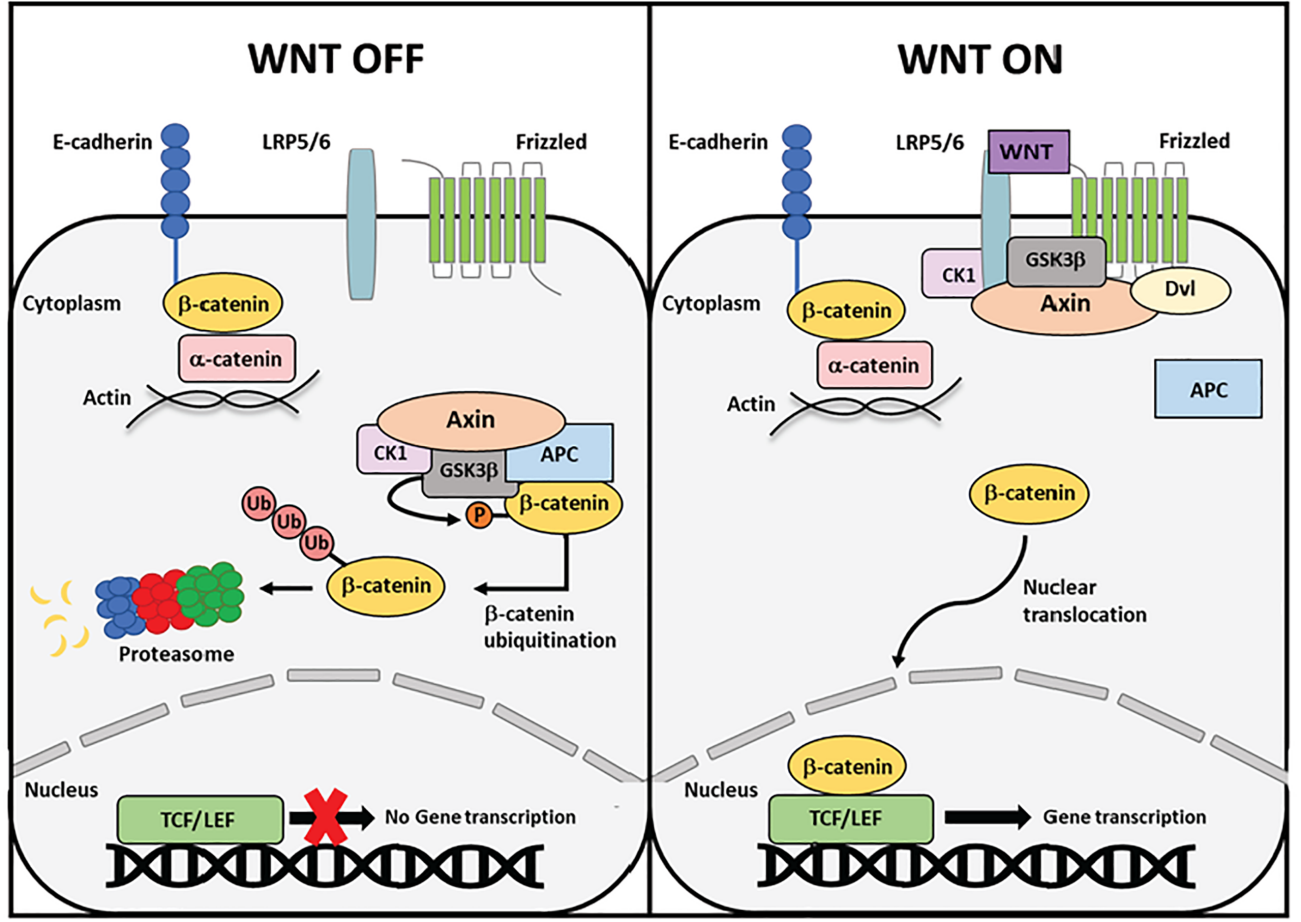
Schematic overview of canonical Wnt signaling. In the absence of a Wnt ligand, cytoplasmic β-catenin is phosphorylated in a multi-protein destruction complex comprised of scaffolding proteins Axin and APC and kinases GSK3β and CK1. Phosphorylation by the destruction complexes induces poly-ubiquitination of β-catenin and subsequent degradation by the proteasome. Upon Wnt ligand binding to the Frizzled receptor and LRP5/6 co-receptor, proteins in the destruction complex are recruited to the cell membrane, rendering the complex inoperative. Cytoplasmic β-catenin can now escape degradation and translocate to the nucleus, where it binds the TCF/LEF family of transcription factors and initiates target gene transcription.

Wnt-signaling is a core developmental pathway important for cell proliferation and migration. β-catenin is a critical protein for canonical Wnt-signaling. Activation of Wnt-signaling allows β-catenin to evade the negative regulation of a cytoplasmic destruction complex promoting β-catenin translocation to the nucleus and activation of Wnt target genes (**Figure 1**). Alterations to β-catenin or destruction complex members are implicated in numerous cancers, namely colon and endometrial cancer (9). However, β-catenin also serves an important function at the adherens junctions, linking E-cadherin at the cell membrane to the actin cytoskeleton through α-catenin. The goal of this review is to examine the function of β-catenin as both a signaling and adhesion protein and explore how mutations may impact endometrial cancer progression.

## β-catenin: History and structure

β-catenin is an evolutionarily conserved protein encoded by the *CTNNB1* gene that was first identified in the late 1980s as a binding partner of uvomorulin, later shown to be E-cadherin (10). Immunoprecipitation experiments uncovered three independent proteins bound to uvomorulin, subsequently named alpha-, beta-, and gamma-catenin. Concurrently, β-catenin was identified as a homolog of the mammalian protein plakoglobin, which itself is a homolog of the Drosophila Armadillo gene that regulates developmental polarity (11). Additional work in Drosophila identified the polarity gene, Wingless, regulates the levels of Armadillo (12). This finding began to uncover the role of β-catenin as both a signaling protein and a cell adhesion component.

**Figure 2:**
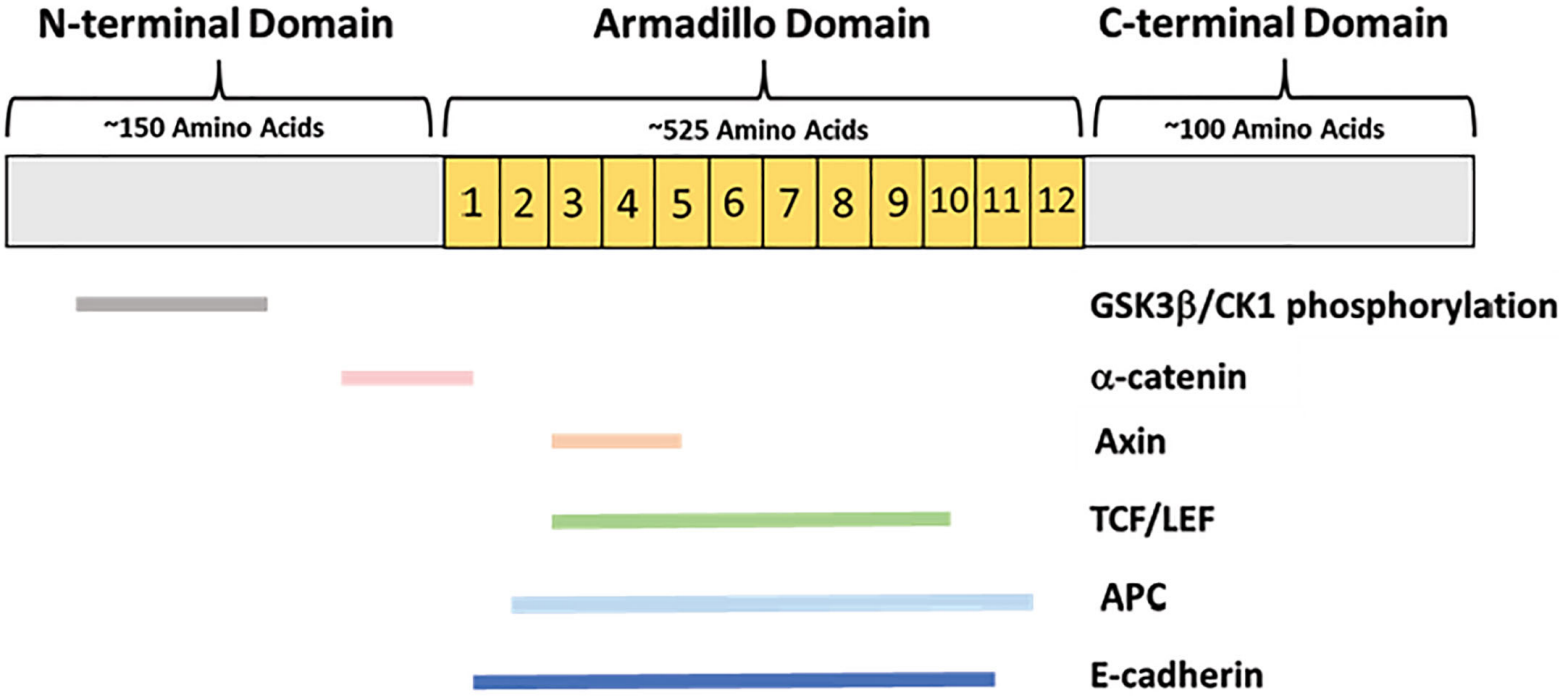
β-catenin protein domains and primary binding partners. β-catenin is comprised of an approximately 150-amino-acid N-terminal domain, a 525-amino acid central Armadillo domain containing 12 Arm repeats, and an approximately 100-amino acid C-terminal domain. The N-terminus is the site of GSK3β and CK1 phosphorylation, as well as partial α-catenin binding. The Armadillo domain contains overlapping binding sites for α-catenin, E-cadherin, Axin, APC, and the TCF/LEF transcription factors.

In higher organisms β-catenin has two primary functions, in adherens junctions and as a member of the canonical Wnt-signaling pathway. The structure of β-catenin enables multiple functions within the cell (**Figure 2**). In 1996 it was discovered that β-catenin can conduct both signaling and adhesion functions through partner binding to separate regions of the protein (13) (**Figure 2**). The β-catenin protein is comprised of three domains: an N-terminal domain, a C-terminal domain, and a central domain comprised of 12 Armadillo (Arm) repeats. The N-terminus contains binding sites for proteins within a destruction complex that signals β-catenin for degradation. The C-terminus end serves as a binding site for members of the TCF/LEF family of transcription factors in the nucleus (14). Binding partners for both the N-terminus and C-terminus are important for Wnt-signaling function (**Figure 2**). The central Arm repeats form a highly conserved region sharing homology with other Armadillo family proteins. This region is made up of 12 repeating segments that form a super-helix structure with a large positively charged groove. The Arm region is the site of E-cadherin binding making this area critical for β-catenin function at the adherens junctions. Each region of the β-catenin protein allows for a unique set of binding partners to carry out numerous functions in the cell (**Figure 2**).

Beyond protein structure, specific amino acid residues also regulate the multiple functions of β-catenin. At the adherens junctions, E-cadherin interacts with β-catenin throughout most of the Arm region, but the core binding regions are Arm repeats 5-9 and 11-12. Within repeats 5-9 are amino acids K312 and K435, which are necessary for E-cadherin binding (15). α-catenin binds β-catenin primarily in the first Arm repeat. This binding interaction disrupts the Arm repeat near Y142 and D144 to create a hinge where both α-catenin and E-cadherin can bind β-catenin at the same time. One residue important for β-catenin regulation at the adherens junction is Y654. Phosphorylation of Y654 by Src obstructs E-cadherin binding and greatly reduces the affinity of E-cadherin for β-catenin (16).

Within the N-terminus, the stretch of amino acids 32-45 contains residues critical for β-catenin regulation in Wnt-signaling. S45 is phosphorylated by CK1 priming this region for phosphorylation of S33, S37, and T41 by GSK-3β. Phosphorylation by CK1 and GSK-3β are required prerequisites for ubiquitination and therefore regulate β-catenin levels in the cytoplasm (17). In addition to the N-terminus, phosphorylation in the C-terminus and Armadillo domain is important for β-catenin stabilization and activation of transcription. S675 can be phosphorylated by protein kinase A (PKA), inhibiting ubiquitination thereby stabilizing β-catenin (18). Additionally, phosphorylation of S552 by AKT can divert β-catenin from the adherens junctions to the cytoplasm and nucleus where it can bind to 14-3-3 ζ and increase transcriptional activity (19).

## β-catenin: Duality of functions

Another important determinant of β-catenin functionality is protein localization within the cell. β-catenin is localized to the plasma membrane when bound to E-cadherin at the adherens junctions. Additionally, β-catenin resides in the cytoplasm where it can translocate to the nucleus during Wnt-signaling (**Figure 1**). These functions are seemingly independent of one another, as they occur by different binding interactions and subcellular locations (20).

Adherens junctions are cadherin- and catenin-based junctions that promote cell-cell adhesion. Adherens junctions are present on polarized epithelia and are important for cells to respond to extracellular signals and changes in force. They are dynamic junctions that form and re-form in response to extracellular signals. At the membrane, β-catenin is bound to the cytoplasmic tail of E-cadherin, a Ca2+ dependent transmembrane protein expressed in epithelial cells (21). The extracellular portion of E-cadherin can associate with other cadherins on adjacent cells. E-cadherin bound β-catenin binds α-catenin in the cytoplasm linking the AJ to the actin cytoskeleton of the cell. β-catenin and E-cadherin associate in the endoplasmic reticulum after being synthesized and then translocate together to the plasma membrane (22). Once at the membrane β-catenin and α-catenin can associate together with the actin cytoskeleton.

The other important function of β-catenin is in the Wnt-signaling pathway (**Figure 1**). Wnt-signaling is a developmental pathway important for establishing polarity in embryogenesis, inducing stem cell differentiation, and regulating adult cell proliferation and migration (23). Wnt-signaling can proceed through the canonical or non-canonical pathway. β-catenin functions in canonical Wnt-signaling to regulate gene transcription (24). The Wnt-signaling cascade is turned on by the Wnt family of secreted glycoproteins that contains 19 mammalian members. In the absence of a Wnt-signal, the pool of β-catenin in the cytoplasm is regulated by a multiprotein destruction complex (25). The destruction complex is comprised of many proteins, but the foundational members are Axin, Adenomatous Polyposis Coli (APC), Glycogen synthase kinase-3-beta (GSK3β), and casein kinase 1 (CK1). Axin is a large scaffolding protein upon which other destruction complex proteins can dock including the serine/threonine kinases GSK3β and CK1. Unbound β-catenin in the cytoplasm is targeted by the destruction complex. CK1 first “primes” β-catenin by phosphorylating S45. Next, GSK3β can phosphorylate S33, S37, and T41. Phosphorylation at the N-terminus of β-catenin creates a docking site for β-TRCP1, a member of the SCF family of E3 ubiquitin ligases. This E3 ligase recruits an E2 ligase, which polyubiquitinates β-catenin at K19 and K49, and leads to subsequent protein degradation by the proteasome (25). In this way, levels of β-catenin in the cytoplasm are kept consistently low in the absence of Wnt signaling (**Figure 1**).

When the Wnt-signaling pathway is turned on, Wnt ligand binds to the Frizzled (Fz) receptor at the cell membrane. The G-protein coupled receptor Fz is a seven transmembrane protein that is linked to the low-density-lipoprotein-related protein 5/6 (LRP5/6) co-receptors to carry out canonical Wnt-signaling. Upon ligand binding to Fz, the Disheveled (Dvl) phosphoprotein is recruited from the cytoplasm to bind the Fz receptor. The formation of this complex causes dissolution of the destruction complex by recruiting the Axin scaffolding protein to the plasma membrane where it docks on the cytoplasmic tail of the LRP5/6 co-receptor. After Axin docking, LRP5/6 can be phosphorylated by CK1 or GSK3β, further strengthening the destruction complex recruitment to the membrane (23). Once the destruction complex is disassembled and recruited to the membrane, cytoplasmic β-catenin is no longer targeted for degradation (23). Undegraded β-catenin can accumulate in the cytoplasm and translocate to the nucleus without restrictions. In the nucleus, β-catenin binds to and activates the T-cell factor/lymphoid enhancing factor (TCF/LEF) family of transcription factors. TCF/LEF bind β-catenin along Arm repeats 3-10, where K312 and K435 are necessary for binding to TCF/LEF (26). Without β-catenin, TCF/LEF are complexed to members of the Groucho/TLE family of transcriptional co-repressors. β-catenin displaces Groucho/TLE in the nucleus and binds TCF/LEF to activate transcription. Canonical Wnt-signaling induces the transcription of different target genes depending on the tissue type. Notable target genes are *CCND1*, which encodes the Cyclin D1 cell cycle protein, and *c-Myc* and *Jun*, which are both proto-oncogenes (27).

The exact mechanism by which β-catenin nuclear translocation occurs is not well understood, as β-catenin does not contain a nuclear localization signal (NLS) (28). Nuclear import and export are often facilitated by soluble transporter proteins like importins and exportins. Members of the importin-β family can directly bind cargo through its NLS with the help of importin-α (29). Loading and unloading of cargo are regulated by the GTPase Ran, where RanGTP levels are low in the cytoplasm and high in the nucleus to enable directional transport (30). However, it has been found that β-catenin can enter the nucleus in a RanGTP independent manner (31). Interestingly, the Arm repeats of β-catenin are structurally similar to the HEAT repeats of importin-β (32), therefore it has been proposed that β-catenin may possess some independent transport abilities.

The questions of what prompts β-catenin to translocate from the cytoplasm to the nucleus remain. One proposed mechanism involves Rac1, a member of the Rho family of small GTPases. A previous study suggested a signaling cascade in which Wnt family member Wnt3a activates Rac1, in turn prompting c-Jun N-terminal kinase 2 (JNK2) to phosphorylate β-catenin at S191 and S605 inducing nuclear translocation (33). Further, a more recent study suggests that Wnt-mediated Rac1 activation not only induces nuclear translocation, but also enhances β-catenin and LEF-1 binding in the nucleus (34). Another proposed mechanism indicates that β-catenin nuclear translocation is influenced by its binding partners. Studies have suggested either Axin or APC as a molecular chaperone to shuttle β-catenin from the cytoplasm to the nucleus (35, 36). Additionally, α-catenin has been found to have a nuclear function. Several studies have shown that the nuclear localization and function of α-catenin is dependent on nuclear β-catenin (37, 38). Therefore, α-catenin may be mutually required for β-catenin nuclear translocation. Nevertheless, the exact mechanism driving nuclear translocation of β-catenin remains unclear.

The Wnt-signaling and adherens junction functions of β-catenin are seemingly independent, as evidenced by competition between binding partners. E-cadherin, APC, and TCF/LEF transcription factors all bind the central Arm repeats of β-catenin (**Figure 2**). Association with E-cadherin in the endoplasmic reticulum may be protective against cytoplasmic β-catenin being degraded by the destruction complex (39). Previous studies have shown that E-cadherin can compete with both APC and LEF-1 to bind β-catenin (39, 40). In a set of immunoblotting and affinity precipitation experiments, E-cadherin, APC, and LEF-1 were found to form independent, but competitive complexes with β-catenin. Additionally, a greater amount of β-catenin was localized to the cytoplasm or nucleus in E-cadherin deficient cells (39). In the absence of E-cadherin, there was more interaction between β-catenin and LEF-1 than in E-cadherin wild-type cells. This suggests that the localization of β-catenin is greatly determined by the presence or absence of E-cadherin.

## *CTNNB1* mutations and endometrial cancer

Alterations to Wnt-signaling proteins and mutations in *CTNNB1* are associated with multiple types of cancer. Additionally, loss of E-cadherin at adherens junctions is a critical step in the cellular process of the epithelial-to-mesenchymal transition (EMT). EMT is a process by which epithelial cells lose their characteristic cell-cell adhesion, polarity and transition into more migratory mesenchymal cells. This process is important for normal development, but also enables cancer cells to invade through the basement membrane promoting metastasis (41). Therefore, the cell adhesion and Wnt-signaling functions of β-catenin can be implicated in cancer progression.

**Figure 3:**
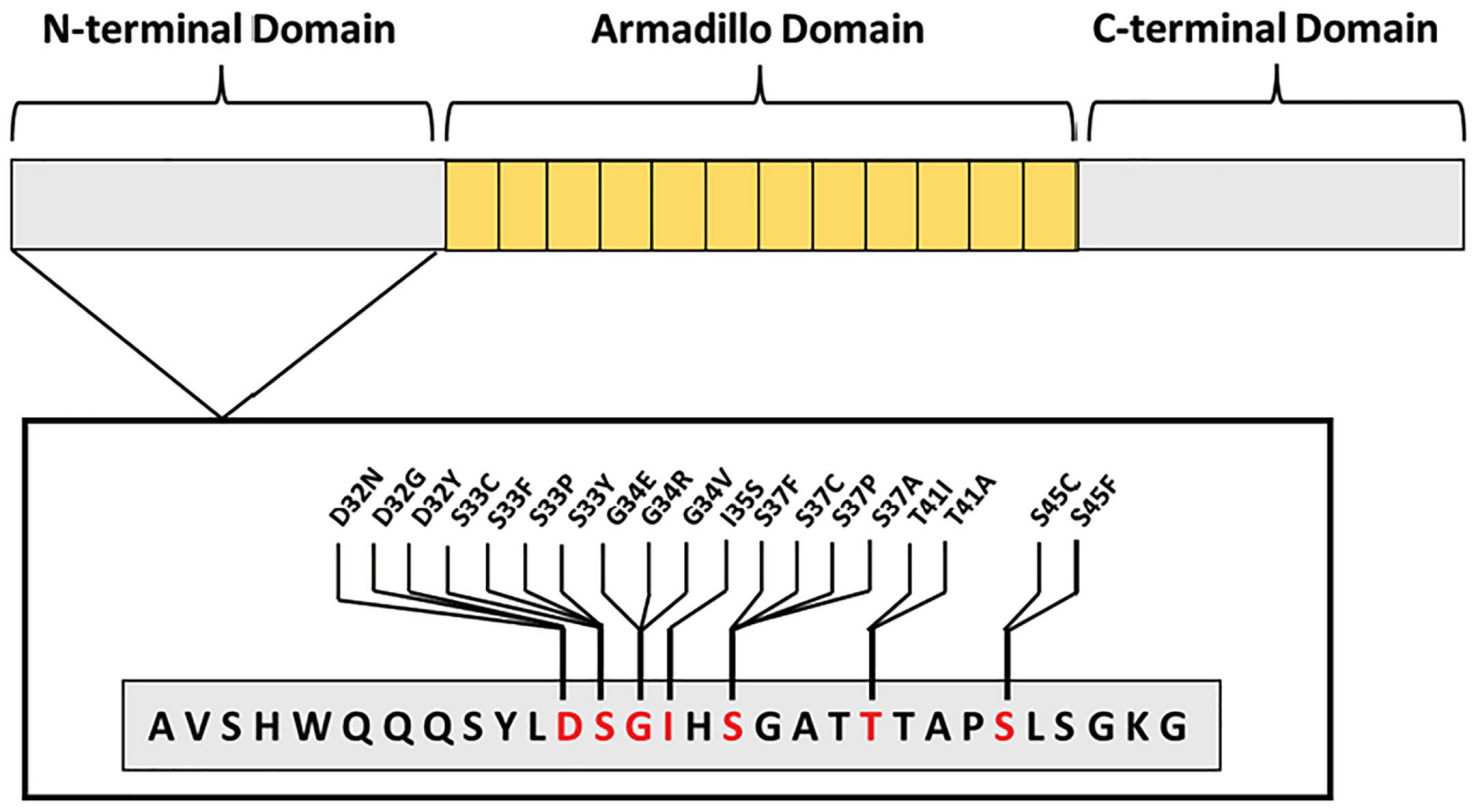
Most commonly mutated β-catenin amino acid residues occurring in endometrial cancer. All mutations occur in exon 3, found within the N-terminus, blocking GSK3β or CK1 phosphorylation sites—S33, S37, T41, S45—or adjacent residues—D32, G34, and I35.

A large sequencing study of cancer patients revealed *CTNNB1* mutations were most commonly observed in endometrial, liver, and colorectal tumor types, with endometrial cancer being the most prevalent (42). The majority of these *CTNNB1* gene alterations occur in the N-terminal exon 3 region. This region notably contains the phosphorylation sites for GSK3β and CK1 (**Figure 3**). In endometrial cancer, patients with *CTNNB1* mutations typically have missense mutations at phosphorylation sites and/or adjacent residues (**Figure 3**). These exon 3 mutations in endometrial cancer are thought to protect β-catenin from degradation by the destruction complex. Mutant β-catenin can accumulate in the cytoplasm and translocate to the nucleus without regulation. Consequently, TCF/LEF transcription factors can be constitutively activated. Because canonical Wnt-signaling induces transcription of genes regulating cell cycle induction and proliferation, this overactive Wnt-signaling can contribute to cancer progression.

The subset of patients with *CTNNB1* mutations almost exclusively occur in endometrioid endometrial cancer (EEC). The endometrioid histological type is the most common, with 70-80% of patients having EEC. However, this large group of tumors are incredibly heterogeneous. A multivariate analysis of 271 EEC patient’s data from The Cancer Genome Atlas (TCGA) was conducted to better classify EEC tumors (43). This study identified four clusters (I, II, III, IV) with distinct molecular profiles. Notably, 87% of tumors in Cluster II harbored *CTNNB1* mutations, with most being exon 3 missense mutations. The majority of mutations were at phosphorylation sites S33, S37, T41, S45 or adjacent residues D32 and G34 (**Figure 3**). Cluster II comprises approximately 28% of all EEC cases analyzed in this study. Further, Gene Set Enrichment Analysis of this patient cluster revealed an association between exon 3 *CTNNB1* mutations and overexpression of other Wnt-signaling proteins. This cluster of patients is notable because it distinguishes EEC patients with *CTNNB1* mutations as a distinct group. EEC tumors typically are low stage and low grade with a favorable prognosis. Both Cluster I and Cluster II in this study are comprised of obese patients with low grade and low stage tumors. However, Cluster II is notable because these patients are younger in age and exhibit a worse overall survival rate than patients in Cluster I (43). This study suggests that *CTNNB1* mutations and alterations in other Wnt-pathway proteins may serve as a clinical marker for treatment course or patient outcome.

More recent studies have also identified a prognostic role of exon 3 *CTNNB1* mutations in EEC. A 2019 study by Imboden et al. sought to determine histopathologic and genetic determinants of recurrence in early stage and low grade EEC patients to improve treatment decisions (44). Results of this study found *CTNNB1* to be the most commonly mutated gene in all early-stage and low-grade patients, including non-recurrent, recurrent, and secondary lesions. Although this study found no correlation between recurrence and exon 3 *CTNNB1* mutation, they concluded that the presence of *CTNNB1* mutations in early-stage disease combined with disease recurrence observed in other studies confers a prognostic value for these mutations.

A 2022 study by Travaglino et al. conducted a systemic review and meta-analysis of all studies examining the prognostic value of *CTNNB1* mutations in early-stage EEC (45). Following study selection, 7 studies were included comprising 1,031 early-stage EEC patients with exon 3 *CTNNB1* mutations. Out of 149 patients assessed for recurrence, 44 patients had recurrent disease (22.7%) and 23/44 recurrent patients harbored a *CTNNB1* mutation (52.3%). The significance of recurrence and mutation state increased following exclusion of patients with known molecular status other than the copy-number low/no specific molecular profile (NSMP) TCGA group. Additionally, 886 patients were evaluated for disease-free survival (DFS), of which the molecular status was known for 546. Initial analysis showed no association between *CTNNB1* mutation status and DFS, but exclusion of patients with known molecular status other than the NSMP group caused a significant association between *CTNNB1* mutation and decreased DFS. This study found an overall association between recurrence and DFS in patients with exon 3 *CTNNB1* mutations.

Given the prognostic value of exon 3 *CTNNB1* mutations in EEC, some have suggested a modification to the TCGA molecular classification of endometrial cancer. The current TCGA classification groups *CTNNB1* mutations into the copy-number low group, also known as NSMP. Studies have suggested creating an additional, fifth molecular TCGA category defined by *CTNNB1* mutations (46, 47). Tumors in the copy-number low molecular group have frequent mutations in *CTNNB1*, *PTEN*, *PIK3CA*, *ARID1A*, and *KRAS* (4). Because the mutations present in the copy-number low group are broad, the prognostic value of this molecular group is difficult to define. Further, the European Society of Gynecological Oncology (ESGO), the European Society for Radiotherapy and Oncology (ESTRO), and the European Society of Pathology (ESP) published joint guidelines in 2020 for risk stratification of endometrial cancer patients (47). These guidelines are based upon both TCGA molecular subtypes and clinical characteristics like lymph-vascular space invasion (LVSI). Under these guidelines, the copy-number low/NSMP group may fall under the low, intermediate, or high-intermediate risk category depending on the tumor stage or LVSI. For this reason, considering *CTNNB1* mutations as their own molecular group would facilitate a more accurate representation of the risk factor for patients with *CTNNB1* mutations. Further, a 2022 study by Kurnit et al. evaluated the effects of adjuvant therapy on recurrence-free survival in endometrial cancer patients with *CTNNB1* mutations (48). This study also characterized the risk category according to myometrial invasion and LVSI, integrating molecular and clinicopathologic characteristics. Results of this study indicated that patients with *CTNNB1* mutations at intermediate risk, defined as any grade endometrioid cancer with deep myometrial invasion or LVSI, had improved recurrence-free survival following adjuvant therapy. Accordingly, recurrence-free survival of low-risk patients with *CTNNB1* mutations was not impacted by adjuvant treatment. Taken together, these studies suggest that defining endometrial cancers by both molecular characteristics and clinicopathologic features is a more effective way to assess risk factor and treatment options, particularly in the case of *CTNNB1* mutations.

## Context dependence of β-catenin mutations in endometrial cancer

*CTNNB1* mutations in EC represent a patient group with worse recurrence free survival rates (49, 50). However, the magnitude of the impact of *CTNNB1* mutations is context dependent. The majority of *CTNNB1* mutations in EC occur in the endometrioid histological subtype. Though *CTNNB1* mutations can occur in the non-endometrioid histological subtype, they are rare. Further, mutations in *CTNNB1* are associated with worsened survival in patients with low grade, endometrioid type cancers. Therefore, stratifying patient survival data by grade and histological type is critical to unmasking the prognostic importance of *CTNNB1* mutations in EC.

The disparity in survival between *CTNNB1* mutant and wild-type patients can be seen clearly in low grade, endometrioid type ECs. A 2021 study by Caracuel et al. examined the prognostic value of *CTNNB1* mutations in a cohort of 218 patients with low grade and low stage disease (51). Specifically, all patients in this cohort were categorized as primary endometrioid grade 1 or grade 2 ECs. The results of this study showed a significantly decreased disease-free survival (DFS) in patients with exon 3 *CTNNB1* mutations compared to those with wild-type *CTNNB1*. Further, the relationship between *CTNNB1* mutation and DFS was independent of other prognostic determinants such as age and The International Federation of Gynecology and Obstetrics (FIGO) stage. This study highlights the impact of exon 3 *CTNNB1* mutations on DFS in EEC patients.

In addition to survival, exon 3 *CTNNB1* mutations in EEC can be a marker for disease recurrence. A 2020 study by Moroney et al. evaluated a cohort of grade 1, stage 1 EEC cases to identify molecular markers associated with higher recurrence risk (52). 311 women with grade 1, stage 1 EEC were identified, of which 18 had recurrent disease and 30 were selected as matched non-recurrent controls. Molecular testing revealed that 60% of recurrent cases had exon 3 *CTNNB1* mutations whereas 28% of non-recurrent controls had exon 3 *CTNNB1* mutations. These results suggest that *CTNNB1* mutation status can serve as a clinical marker for disease recurrence in low grade and stage EEC. Grade 1 and stage 1 endometrial cancers are considered to be low risk. Because this study uses low risk patients all without adjuvant treatment, confounding variables have been controlled for and the effects of *CTNNB1* mutations on recurrence are more impactful.

Another 2020 study by Costigan et al. sought to correlate β-catenin and Cyclin D1 immunohistochemistry with exon 3 *CTNNB1* mutant EEC cases and assess the clinicopathologic features associated with these patients (53). Within the cohort of 79 patients, 34 harbored exon 3 *CTNNB1* mutations while 45 had wild-type *CTNNB1*. In contrast to the previous studies discussed, the cohort selected for this study contained grades 1, 2, and 3 EEC patients. Differences in survival and recurrence between *CTNNB1* mutant and wild-type EECs are typically observed in low grade cancers only. The present study utilized follow-up data on stage IA patients to assess disease recurrence. They found that 30% of patients with exon 3 *CTNNB1* mutations had disease recurrence whereas no patients with wild-type *CTNNB1* had recurrent disease. Because recurrence rates were defined by a low FIGO stage, all patients analyzed had grade 1 or 2 EEC. Additionally, they found no difference in recurrence rates between *CTNNB1* mutant and wild-type patients presenting with high stage EEC. These results further emphasize the significance of characterizing *CTNNB1* mutant and wild-type survival and recurrence data by grade.

The mutation status of *CTNNB1* is an important clinical marker in low grade, endometrioid type EC. However, the value of this marker can be lost when high- and low-grade EEC patients are grouped together. Studies of survival or recurrence containing cohorts of grades 1-3 EECs or both EECs and non-EECs may lose the impact of exon 3 *CTNNB1* mutations if grade 1-2 cases are not delineated. Exon 3 *CTNNB1* mutations can occur in high-grade EECs and, rarely, in non-EECs. However, a difference in survival and recurrence is not evident in these categories. Therefore, patients with grades 1-2 EEC harboring exon 3 *CTNNB1* mutations must be treated as a separate entity. While independent studies have identified low grade EEC patients with *CTNNB1* mutations as having worsened survival, none of these publications have evaluated survival according to exon 3 *CTNNB1* mutation type. One hinderance to such analysis is the high number of unique reported exon 3 *CTNNB1* mutations per study, with 15 different mutations in one report (53) and 22 different mutations in a subsequent report (54). Future studies examining the relationship between survival and type of exon 3 *CTNNB1* mutations will be valuable to further define the context of these mutations in low grade EEC patients.

## Membrane and cytoplasmic pools of β-catenin and endometrial cancer

In normal epithelial cells β-catenin is primarily localized to the cell membrane associated with E-cadherin. However, β-catenin is often thought of in the context of canonical Wnt-signaling in endometrial cancer. This is due to frequent hotspot mutations at the N-terminus region that preclude the CK1 and/or GSK3β phosphorylation sites. The membrane and cytoplasmic pools of β-catenin function differently and seemingly in an independent manner. Nonetheless, loss of cell-to-cell adhesion by way of the adherens junctions can contribute to uncontrolled cell proliferation and metastasis (55). This begs the question of how *CTNNB1* mutations found in endometrial carcinoma may affect β-catenin at the membrane as well as in the cytoplasm.

A 2001 study compared β-catenin localization in normal, hyperplastic, and endometrioid carcinoma endometrial tissue (5). The immunoreactivity score for β-catenin staining at the membrane was highest for normal samples and decreased steadily through non-atypical hyperplasia, atypical hyperplasia, and grades 1-3 carcinoma lesions. Conversely, no β-catenin nuclear localization was observed in normal samples, very little was seen in non-atypical hyperplastic lesions, and clear nuclear localization was present in atypical hyperplasia and grades 1-3 carcinoma tissues. This data suggests that β-catenin localization changes with an increasing degree of atypical hyperplasia or carcinoma grade. Additionally, they compared endometrial carcinoma cases with or without exon 3 *CTNNB1* mutations and β-catenin staining patterns. Cases with *CTNNB1* mutations had significantly lower amounts of membrane β-catenin staining and higher amounts of nuclear β-catenin staining than cases without *CTNNB1* mutations. It is known that exon 3 mutations cause evasion of the degradation complex and can lead to cytoplasmic accumulation of β-catenin. However, a correlated loss of membrane staining may indicate a change in subcellular distribution of β-catenin following *CTNNB1* mutations. Although exon 3 mutations do not overlap with E-cadherin binding sites, perhaps there is a mechanism by which the hotspot mutations affect β-catenin distribution while still within the endoplasmic reticulum. Further research is warranted to investigate the reciprocal nature of β-catenin membrane and nuclear distribution in endometrial carcinoma.

The majority of exon 3 *CTNNB1* mutations occur in low grade, endometrioid endometrial cancer. Alterations to β-catenin arise early in endometrial cancer pathogenesis. Conversely, alterations to E-cadherin can increase the metastatic potential of cells, which commonly develops only in high grade or high stage endometrial cancer (56). It is possible that *CTNNB1* mutations only affect the cytoplasmic pool of β-catenin because the membrane-bound pool is heavily dictated by E-cadherin. Thus, the dissolution of adherens junctions could rely on changes to E-cadherin, not β-catenin. A 2003 study examined cadherin and catenin levels in 149 endometrial lesions (21 endometrial atypical hyperplasia (AEH), 68 EECs, 27 non-EECs) (57). They found that non-EEC lesions like serous, clear cell, mixed serous-clear cell, or mixed endometrioid-serous types had significantly reduced E-cadherin compared to AEH and EEC lesions. Additionally, they found less membranous β-catenin in lesions with reduced E-cadherin expression than those with wild-type E-cadherin. Alterations to E-cadherin in these lesions reduces the need to inactivate β-catenin directly. Non-EEC carcinomas typically have a higher stage at diagnosis and a poorer prognosis. These results suggest that reduced E-cadherin may negatively affect the amount of β-catenin at the membrane in high grade endometrial cancer. This may be due to the co-translational relationship between E-cadherin and β-catenin in the endoplasmic reticulum.

**Figure 4:**
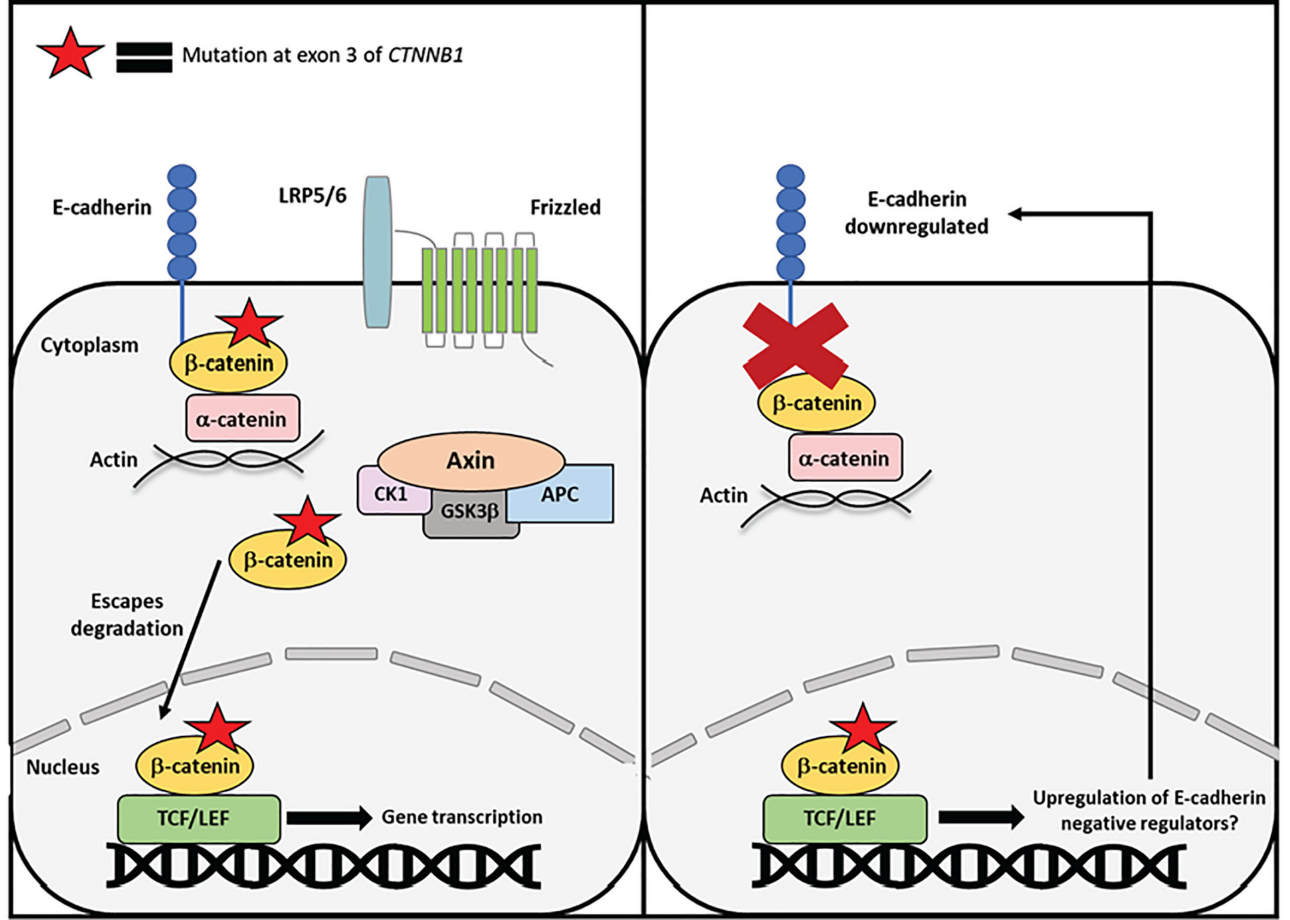
Proposed mechanism of exon 3 CTNNB1 mutations in endometrial cancer. Mutant β-catenin can escape degradation in the cytoplasm and translocate to the nucleus independent of Wnt-signaling. One mechanism by which mutant β-catenin may drive endometrial cancer is by inducing transcription of E-cadherin negative regulators or EMT genes. β-catenin mutations occur relatively early in endometrial cancer progression, whereas loss of E-cadherin occurs later in disease progression. Therefore, mutations to β-catenin may indirectly contribute to E-cadherin loss and EMT progression.

The decreased expression of E-cadherin in endometrial cancer is not fully understood. There are various mechanisms that are thought to be causative, such as loss of heterozygosity and promoter hypermethylation. Another possibility is dysregulation of transcriptional repressors of E-cadherin. The transcription factors Twist, Snail1, Snail2, and Zeb1 all work to repress E-cadherin and are known EMT markers. Further, these transcription factors are all directly or indirectly regulated by β-catenin or Wnt-signaling (58). Thus, exon 3 *CTNNB1* mutations may not impact membranous β-catenin by interfering with E-cadherin binding, but rather indirectly affect adherens junctions by upregulating repressors of E-cadherin (**Figure 4**).

## Clinical applications of *CTNNB1* mutations in endometrial cancer

Treatment regimens for endometrial cancer have remained relatively consistent in recent decades. The primary treatment option is surgery, typically consisting of hysterectomy, salpingectomy, and occasional lymph node dissection. One barrier to treatment is the lack of consensus on treating recurrent endometrial cancer. Radiation therapy is the current standard of care for preventing local recurrence and treating locally recurrent endometrial cancer, but further treatment options are limited (59). Currently, guidelines do not incorporate molecular biomarkers into treatment decisions for preventing recurrent disease. As previously discussed, Kurnit et al. have recently shown that incorporating the status of *CTNNB1* exon 3 mutations helps to identify stage I EEC patients who would benefit most from adjuvant radiation treatment following surgery (48).

Given the poor treatment response of advanced EC to conventional chemotherapy approaches, targeted therapies have emerged as a better treatment option for these patients. Given its role as a prognostic marker, β-catenin and the Wnt-signaling pathway have been implicated as potential targets for endometrial cancer treatment. One promising therapeutic is DKN-01, a monoclonal antibody against Dickkopf-1 (DKK-1). DKK-1 is a negative regulator of Wnt-signaling that functions by binding LRP5/6 and blocking Wnt ligands. Additionally, studies have found that overexpression of DKK-1 in some solid tumors is correlated with worsened survival (60). DKN-01 has been studied in the context of several gynecological and gastrointestinal cancers, which tend to have frequent alterations in Wnt-signaling pathway members. One study examining the effects of DKN-01 treatment or DKK-1 overexpression in endometrioid ovarian cancer (EOC) releveled that overexpression of DKK-1 caused a decrease in immune activity while treatment with DKN-01 did not phenotypically affect EOC cells *in vitro*, indicating that DKN-01 functions by modulating anti-tumor immunity (61). Additional studies have found that DKN-01 reverses the immunosuppressive effects of upregulated DKK-1 in several cancer types and functions by promoting natural killer cells, reducing myeloid-derived suppressor cells (MDSCs), and upregulating PD-L1 on MDSCs (62). DKN-01 may be an effective treatment in combination with other immune modulating treatments for EEC patients with aberrant Wnt-signaling caused by *CTNNB1* mutations. Additionally, DKN-01 has been tested in a phase 2 clinical trial in combination with paclitaxel treatment in epithelial endometrial and ovarian cancer patients (NCT03395080).

Another targeted treatment option for endometrial cancer patients with aberrant β-catenin/Wnt signaling is Porcupine (PORCN) inhibitors. PORCN is an enzyme residing in the endoplasmic reticulum that functions to palmitoleate Wnt ligands post-translationally at conserved serine residues. The palmitoleation of Wnts is important for both Wnt secretion and binding to the Frizzled receptor (63). Inhibition of PORCN is an effective strategy to inhibit overactive Wnt-signaling without targeting β-catenin directly. A 2016 study evaluated the efficacy of a novel, oral PORCN inhibitor, ETC-159 in colorectal cancers (CRC) harboring RSPO-translocations that increase Frizzled and LRP5/6 cell surface supply (64). Results of this study indicated that CRC patients with RSPO2/3 translocations respond well and are highly sensitive to ETC-159. Additionally, global remodeling of gene expression revealed a downregulation in Wnt-pathway target genes in ETC-159 treated tumors. Because *CTNNB1* mutations in EEC constitute an alteration to Wnt-signaling downstream of the Frizzled receptor, PORCN inhibitors may not be efficacious without combination with a molecule targeted to β-catenin specifically. A phase 1A/B clinical trial is ongoing to test the safety and tolerability of ETC-159 as a single agent or in combination with Pembrolizumab in different advanced solid tumors (NCT02521844). Therefore, additional insights are needed to evaluate the utility of ETC-159 and PORCN inhibitors in EEC.

Targeting β-catenin directly in cancer has proved challenging, despite the prognostic value of exon 3 *CTNNB1* mutations in endometrial cancer. PRI-724 is a β-catenin inhibitor that works as an agonist for the association of β-catenin and cyclic AMP response element-binding protein (CBP), a co-activator of transcription (65). Because PRI-724 targets nuclear β-catenin and inhibits subsequent transcription through TCF/LEF, it appears as a promising option for targeting mutant β-catenin in endometrial cancer. Currently, a phase II trial for PRI-724 treatment alone or in combination with chemotherapy and Bevacizumab is ongoing to treat metastatic CRC patients (NCT02413853). CRC has frequent mutations in *APC*, another downstream Wnt-pathway protein, and therefore additional studies of PRI-724 efficacy in EEC patients with *CTNNB1* mutations may be beneficial.

## Conclusion

β-catenin is an evolutionarily conserved protein encoded by the *CTNNB1* gene. It functions both at the cell membrane and the cytoplasm/nucleus in adherens junctions and Wnt-signaling, respectively. These roles are thought of as separate entities due to their distinct locations and binding partners. Mutations in exon 3 of *CTNNB1* are found in a subset of endometrioid endometrial cancer patients and studies have suggested that changes in β-catenin cellular localization in EEC are caused by exon 3 mutations (5). These mutations in endometrial cancer are typically associated with β-catenin nuclear localization. By this mechanism, exon 3 mutations directly affect the Wnt-signaling function of β-catenin by blocking CK1 and GSK3β phosphorylation sites. However, it is not well understood how these mutations may alter the functionality of β-catenin at the adherens junctions. Reduced expression of E-cadherin and β-catenin have been found in non-endometrioid endometrial cancer (56). Both reduced cadherin-catenin expression and unchecked β-catenin translocation to the nucleus can contribute to endometrial carcinoma, albeit in different ways. Similar to their separate cellular functions, perhaps the membrane and cytoplasmic pools of β-catenin are contributing to different types of endometrial carcinoma entirely. Alternatively, β-catenin mutations might affect adherens junctions indirectly by inducing transcription of negative regulators of E-cadherin. Additional research is required to uncover how the separate pools of β-catenin function following exon 3 mutations, and how they might contribute to endometrial cancer pathogenesis.

## Author contributions

MP wrote manuscript and generated figures. RB edited manuscript and contributed insight. AG edited and contributed to manuscript and figures. All authors contributed to the article and approved the submitted version.

## Funding

This work was supported by the NIH SPORE in Uterine Cancer NIH P50 CA098258 and a UNC Lineberger Comprehensive Cancer Center Developmental Award, which is supported in part by P30 CA016086 Cancer Center Core Support Grant.

## Conflict of interest

The authors declare that the research was conducted in the absence of any commercial or financial relationships that could be construed as a potential conflict of interest.

## Publisher’s note

All claims expressed in this article are solely those of the authors and do not necessarily represent those of their affiliated organizations, or those of the publisher, the editors and the reviewers. Any product that may be evaluated in this article, or claim that may be made by its manufacturer, is not guaranteed or endorsed by the publisher.
